# Impact of Surface Finishing on Ti6Al4V Voronoi Additively Manufactured Structures: Morphology, Dimensional Deviation, and Mechanical Behavior

**DOI:** 10.3390/ma17194879

**Published:** 2024-10-04

**Authors:** Chiara Bregoli, Shiva Mohajerani, Jacopo Fiocchi, Mehrshad Mehrpouya, Mohammad Elahinia, Ausonio Tuissi, Laura Maria Vergani, Carlo Alberto Biffi

**Affiliations:** 1 National Research Council, CNR-ICMATE, Via Gaetano Previati, 23900 Lecco, Italy; jacopo.fiocchi@cnr.it (J.F.); ausonio.tuissi@cnr.it (A.T.); 2 Mechanical Engineering Department, Politecnico di Milano, Via La Masa 1, 20156 Milano, Italy; laura.vergani@polimi.it; 3 Mechanical Industrial and Manufacturing Engineering Department, The University of Toledo, 2801 West Bancroft St., Toledo, OH 43606, USA; shiva.mohajerani@rockets.utoledo.edu (S.M.); mohammad.elahinia@utoledo.edu (M.E.); 4 Faculty of Engineering Technology, University of Twente, Drienerlolaan 5, 7522 Enschede, The Netherlands; m.mehrpouya@utwente.nl

**Keywords:** digital image correlation, medical sandblasting, Voronoi lattice structures, dimensional mismatch, LPBF

## Abstract

Additively manufactured medical devices require proper surface finishing before their use to remove partially adhered particles and provide adequate surface roughness. The literature widely investigates regular lattice structures—mainly scaffolds with small pores to enhance osseointegration; however, only a few studies have addressed the impact of surface finishing on the dimensional deviation and the global and local mechanical responses of lattice samples. Therefore, the current research investigates the impact of biomedical surface finishing (i.e., corundum sandblasting and zirconia sandblasting) on Voronoi lattice structures produced by laser powder bed fusion (LPBF) with large pores and different thicknesses on the surface morphology and global and local mechanical behaviors. MicroCT and SEM are performed for the assessment of dimensional mismatch and surface evaluation. The mechanical properties are investigated with 2D digital image correlation (DIC) in quasi-static compression tests to estimate the impact of surface finishes on local maps of strain. In the quasi-static tests, both the global mechanical performances, as expected, and local 2D DIC strain maps were mainly affected by the strut thickness, and the impact of different surface finishings was irrelevant; on the contrary, different surface finishing processes led to differences in the dimensional deviation depending on the strut thickness. These results are relevant for designing lattice structures with thin struts that are integrated into medical prostheses that undergo AM.

## 1. Introduction

Additive manufacturing (AM) allows for the realization of an irregular lattice structure, the struts’ arrangement of which mimics that of bone trabeculae [1,2] and may improve osseointegration [3]. Recently, medical standard regulations and surgeons have accepted and promoted the use of AM in medical devices and patient-matched prostheses for which longer osseointegration is strongly desirable [4,5,6]. However, studies are still addressing the main issues related to the use of AM in the realization of complex and personalized devices. While research about scaffolds with high density and small pores is relatively widespread, the study of scaffolds presenting a large fraction of pores should be deepened [7,8,9]. The latter type of lattice structures could be inserted into orthopedic prostheses, such as hip stems, to improve osseointegration and to provide proper mechanical support [4,10]. Then, the lattice structure can have a coating or structural purpose. In the former case, the struts usually exhibit a stochastic spatial arrangement, and no specific or local evaluation of the strut dimensions is usually performed since it is the intrinsic stochastic structure that promotes bone ingrowth [11,12]. In the latter case, the struts must provide mechanical support, and therefore, an assessment of the dimensional deviation among real struts’ thicknesses in comparison to those developed using computer-aided design (CAD) is required [10]. Indeed, the AM process may lead to dimensional deviation between the thicknesses of the designed struts and the fabricated ones [13]. Among lattice structures with a large fraction of pores and a load-bearing purpose, the porosity may range between 70% and 90% [14], while strut thickness can be close to that of the bone trabeculae and range between 0.5 and 1 mm [13]. To the best of the authors’ knowledge, the literature deals mainly with samples showing large dimensions of struts (e.g., diameters > 1 mm) [15,16], and very few works address samples with thinner struts (e.g., diameters < 1 mm) [17,18,19].

It is worth observing that smaller strut dimensions should be evaluated by considering the constraints of the specific AM technology employed. 

Besides their design and the possibility of including complex structures in medical devices to enhance osseointegration, the surface of parts fabricated by AM has to be investigated: surface finishing is often necessary to remove partially adhering powder particles left over by the process and fine-tune the surface roughness [20]. However, the more complex the arrangement of the lattice structures, the more different the resulting surface finish is. It is evident that the surface morphology of lattice structures is highly influenced by the inclination of the struts and the building direction with respect to the building platform. Moreover, it is well known that surface finishing processes affect the surface roughness and surface morphology. However, the effect of the same surface finishing process on different starting conditions must be carefully considered. Indeed, similar surface finishing processes may lead to different surface roughness, as reported in [6,21,22].

The global improvement in the fatigue mechanical performance of lattice structures fabricated by AM enabled by surface finishing is often addressed in the literature [23,24,25]. For example, Yanez et al. [24] investigated the fatigue behavior of gyroid structures fabricated by AM and stated that a high surface roughness contributed to crack initiation. Oosterbeek et al. [23] studied the chemical surface treatment of Ti6Al4V lattice structures and demonstrated that Hirtisation was an effective method for reducing surface roughness in porous lattice structures. Nevertheless, the dimensional mismatch between finished parts fabricated by AM and CAD models has not been addressed so far, and no investigation of the local mechanical behavior of single struts has been reported. Indeed, the relationship between post-processed surfaces fabricated by AM in lattice structures, the local mechanical behavior of struts, and the dimensional deviation observed after different surface finishes is rarely addressed in the literature.

The current research aims to assess both the AM processability of bone-like lattice structures with thin struts (diameters ranging between 0.5 mm and 0.9 mm) and the effect of different biomedical surface finishes on similar complex structures, with the aim of providing useful indications applicable to prosthesis fabrication. Indeed, since considerable research efforts are being made to introduce complex lattice structures in medical devices, and considering the above-mentioned points, it is crucial to be aware of the effect of surface finishing on the dimensional deviation of devices fabricated by AM and to elaborate possible strategies to minimize the mismatch between the CAD part and the fabricated sample.

The present research work deals with Voronoi lattice-based samples designed with a stochastic arrangement of struts, which present three constant thicknesses (0.5 mm, 0.7 mm, and 0.9 mm). Such structures were fabricated in Ti6Al4V ELI by laser powder bed fusion (LPBF), heat-treated, and subjected to different biomedical surface finishing processes: corundum-sandblasting and zirconia-sandblasting. The as-built samples were considered for comparison. Initially, similar surface conditions were obtained for disc samples fabricated with perpendicular and parallel building orientations, XZ and XY, respectively. Once the surfaces of the post-processed discs (2D geometry) were assessed, a similar analysis was performed on Voronoi lattice-based structures (3D geometry). Firstly, micro-computed tomography (μ-CT) was employed on the lattice structures for volumetric and surface characterization and for the assessment of the dimensional deviation between the fabricated samples with different surface finishes and the CAD models. Secondly, quasi-static compression tests were performed for the measurement of the global mechanical performances of the samples. Finally, 2D digital image correlation (DIC) was used to evaluate the local strain distributions arising under static compression in differently oriented struts of Voronoi lattice samples subjected to different surface finishing processes. 

## 2. Materials and Methods

### 2.1. Sample Fabrication and Post-Processing

An LPBF system (mod. AM400 from Renishaw, Wotton-under-Edge, Gloucestershire, UK), equipped with a pulsed wave laser was used to fabricate Ti6Al4V ELI (extra-low interstitial) specimens with optimized process parameters in order to reach relative densities of about 99.7% [4,26]. Gas-atomized spherical powder with D_10_ = 20 µm and D_50_ = 34 µm was used. According to the standard related to Ti6Al4V ELI [27], the oxygen content was below 0.13% (equal to 0.07%).

**Figure 1:**
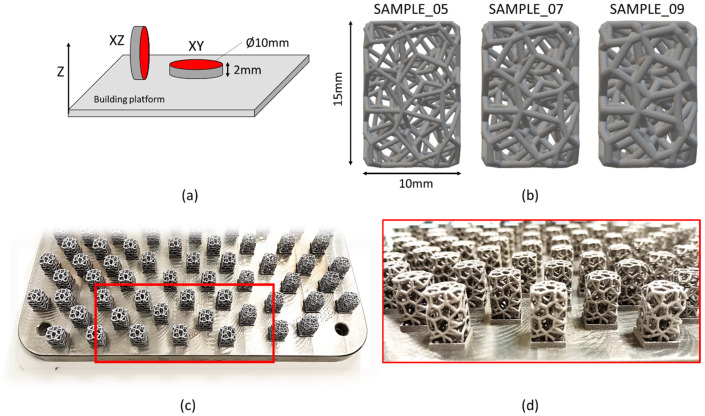
Schematic of the building platform with XZ and XY discs. The red boxes indicate the surfaces analyzed (**a**). CAD models of the three Voronoi lattice-based structures with strut thicknesses of 0.5 mm, 0.7 mm, and 0.9 mm, respectively. The starting full volume was equal to a solid of 10 × 10 × 15 mm (**b**). The building platform with Ti6Al4V ELI LPBFed samples (**c**). The detail of the as-built Voronoi lattice samples (**d**).

Firstly, the disc samples lying either parallel (XY) or perpendicular (XZ) to the building direction were realized (Figure 1a). 

Then, a solid cylinder with a height of 15 mm and a base of 10 × 10 mm was designed as the starting volume to be filled with the Voronoi tessellation for the design of the Voronoi lattice-based structure [28,29]. The Voronoi tessellation has recently attracted interest thanks to its struts arrangement, which is capable of mimicking that of bone trabeculae [30,31]. In fact, just as bone texture is made up of trabeculae arranged in a stochastic disposition, the same occurs in Voronoi tessellation [32]. The designed trabecular samples shared the same 3D spatial arrangement but differed in strut thickness: sample_05, sample_07, and sample_09 exhibited strut thicknesses equal to 0.5 mm, 0.7 mm, and 0.9 mm, respectively (Figure 1b). The average CAD porosity values were 90%, 80%, and 70% for sample_05, sample_07, and sample_09, respectively. These porosities are compatible with that required for biomedical prostheses [33,34]. For each sample, the average CAD porosity was measured as follows:

(1) $$Average\;CAD\;porosity\;\left[\%\right]=\frac{Void\;volume}{Solid\;volume}\times 100$$

The discs and the lattice specimens were subjected to heat treatment at 850 °C for 1 h under vacuum and then naturally cooled at room temperature. An ultrasonic bath was used to clean the samples.

The XZ and XY samples specimens were post-processed with two conventional biomedical surface finishings (Al Ti Color srl, Treviso, Italy): corundum sandblasting and zirconia sandblasting. Conventional parameters were used for each post-processing. 

The Voronoi lattice-based samples were sandblasted with the same biomedical post-processing adopted for the discs to be compared with the as-built surface condition. Three replicas for each condition were obtained (Table 1).

### 2.2. Surface and Volumetric Qualification

**Figure 2:**
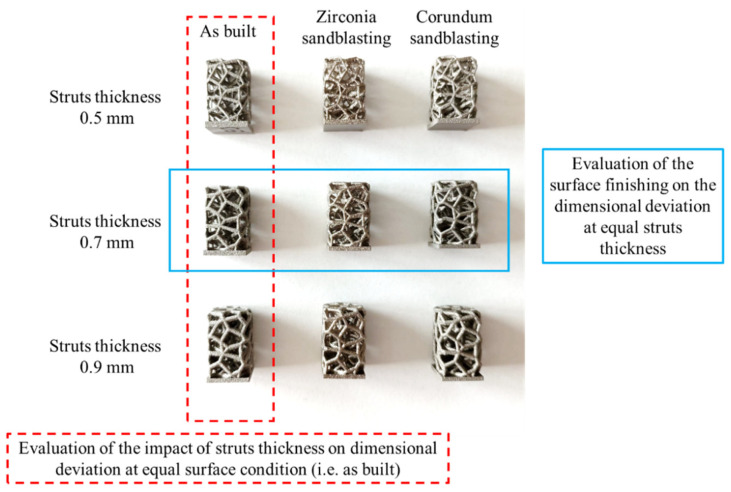
Schematic representation of the 9 investigated conditions. The red box shows the samples subjected to μ-CT analysis, while the blue box shows the samples subjected to profilometric analysis.

Three-dimensional non-contact confocal profilometry (Sensofar S-neox, vertical resolution of 1.5 nm) was employed for the measurement of the surface roughness on different areas (200 × 300 µm^2^) of the observed samples [35]. The profilometry images were then compared to those obtained by scanning electron microscopy (SEM Leo 430 by Zeiss, Oberkochen, Germany). The Voronoi samples were analyzed by SEM to observe the morphologies and surfaces of the struts with different printing orientation. Then, μ-CT scans were performed using a ZEISS system (Xradia Context MicroCT) with a current of 126 mA, an exposure time of 0.75 s, and a voxel size resolution of 16 μm^3^. The obtained tomography datasets were reconstructed using Dragonfly software (Dragonfly v2022.2, Comet Technologies Canada Inc., Montreal, QC, Canada) for the evaluation of the dimensional mismatch between CAD models and fabricated samples. Specifically, the impact of the strut thickness on the dimensional deviation under equal surface conditions was assessed (e.g., as-built) and the effect of the surface finishing on the dimensional deviation at an equal strut thickness (e.g., 0.7 mm with the surface in the as-built condition, zirconia-sandblasted, and corundum-sandblasted) was evaluated (Figure 2). The codification of the samples is reported in Table 1.

### 2.3. Global and Local Mechanical Testing

**Figure 3:**
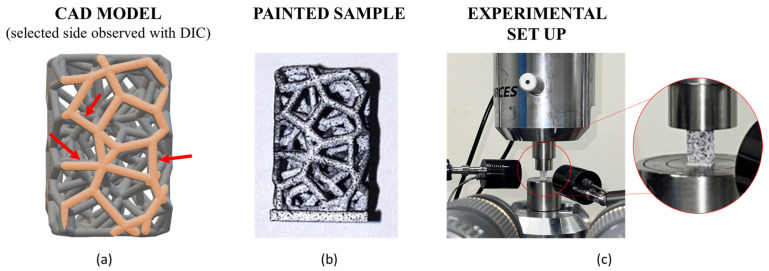
Selected region of interest (ROI) on the CAD model to be observed with DIC. Red arrows indicate struts at different orientations (**a**); painted sample—white plus black dots (**b**); experimental set up for quasi-static compression tests and 2D DIC analysis (**c**).

The sample preparation required for 2D-DIC analysis consisted of creating a random black speckle pattern with a white background on the selected side (i.e., region of interest) of the samples (Figure 3) [36]; the strain field was mapped using the VIC-2D program by Correlated Solutions, with a resolution of 5 megapixels and a frame rate of up to 0.2 Hz. DIC tests were carried out for each of the nine conditions reported in Figure 2. A specific region of interest (ROI) containing vertical, horizontal, and tilted struts (45°) was selected in order to evaluate the local strain maps at varying strut inclinations, thicknesses, and surface finishes.

Then, compression tests were conducted at a strain rate of $5\times 10^{-4}\;s^{-1}$ according to ASTM E9 [37] up to 8% of the strain (1.2 mm of displacement), before the failure of any strut. 

## 3. Results and Discussion

### 3.1. LPBF Process Qualification

The quality of the LPBFed Voronoi lattice-based structures was assessed through both surface and volumetric analyses. Additionally, the impact of the strut thickness on their processability and building accuracy was considered. Higher thickness was expected to be related to a lower warping of the strut walls: however, for proper qualification of the process accuracy, a detailed evaluation of the dimensional deviation was performed using volumetric μ-CT reconstructions. In particular, the processability of thin struts (0.5 mm up to 0.9 mm) with different orientations in a Voronoi lattice-based structure was assessed by μ-CT. 

**Figure 4:**
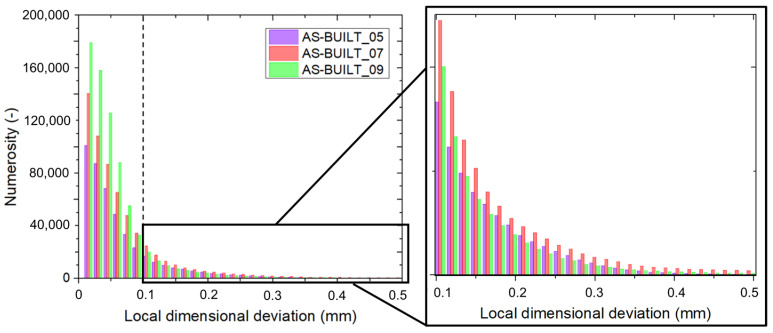
Histogram of dimensional deviation in the as-built samples. The dash line indicates the dimensional deviation of 0.1 mm. Close-up of the region with local dimensional deviation > 0.1 mm.

In the as-built condition, a greater strut thickness was found to be related to a large number of points presenting a local dimensional deviation lower than 0.1 mm (AS-BUILT_09, green bar in Figure 4). On the contrary, the AS-BUILT_05 sample exhibited a lower number of points in which the local dimensional deviation was smaller than 0.1 mm (close-up in Figure 4). 

However, when the local dimensional deviations greater than 0,1 mm were considered, the AS-BUILT_05 presented more deviations (red bar in Figure 4). In summary, larger struts present more points, among which the dimensional deviation is small. Conversely, thinner struts are characterized by a higher share of points, among which the dimensional deviation is large. 

**Figure 5:**
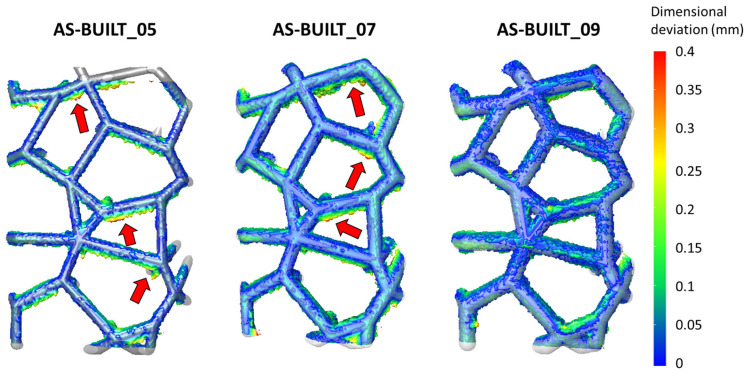
Dimensional deviations in the as-built samples with different strut thicknesses. The AS-BUILT_09 sample exhibited a mainly blue map, which is related to small dimensional deviations. The AS-BUILT_05 sample showed more points among which the powders fell, as expected on the lower skin surfaces (indicated by red arrows). This effect was less evident in the AS-BUILT_09 sample.

As anticipated in the Section 2, 2D DIC observation was performed on a selected ROI (Figure 3), and the dimensional deviation was then quantified at the same ROI (Figure 5). 

The AS-BUILT_05 sample (thinner struts) presented several points among which the dimensional deviation was large (points indicated by red arrows). On the contrary, the AS-BUILT_09 sample did not exhibit points among which the dimensional deviation was bigger than 0.1 mm. On the other hand, the AS-BUILT_09 sample’s surface showed many points among which the dimensional deviation was lower than 0.1 mm. Only a few agglomerations of powders were evident at some nodes in the bottom region of the sample.

Regarding the effect of the different building orientations of the struts, the more tilted struts (angle less than 45 degrees) presented greater dimensional deviation in the samples with thin struts. This was less evident in the samples with larger strut thicknesses (AS-BUILT_09 sample). Indeed, the same struts with similar orientations exhibited high dimensional deviation (>0.1 mm) in the AS-BUILT_05 sample, while the deviation was minimal (<0.1 mm) in the AS-BUILT_09 sample.

Regardless of the strut thickness, struts oriented primarily along the axis of the specimens (*z* axis) revealed the lowest dimensional deviation (Figure 5). 

Finally, it is possible to state that a challenging strut orientation (e.g., strut axis angle < 45 degrees) can be better achieved in AM by increasing the thickness of the struts.

### 3.2. Post-Processing on LPBFed Samples 

Firstly, the selected surface finishes were applied to simple geometries, such as discs built parallel (XY) or perpendicular (XZ) to the building platform. Both orientations were studied to consider the effect of the two extreme surface conditions obtainable by LPBF.

**Figure 6:**
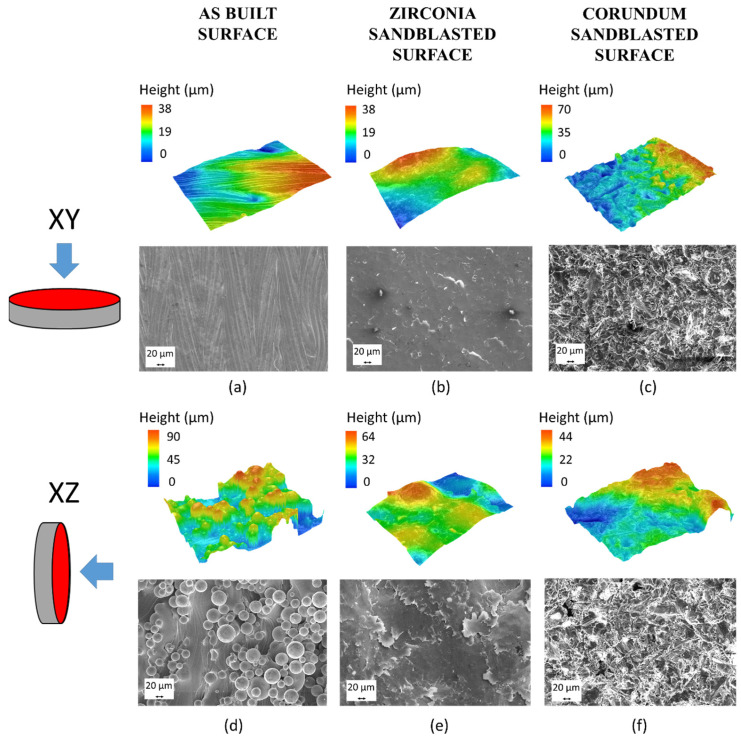
Profilometry and SEM images of the disc samples: representing the two extreme conditions of LPBFed surfaces: as-built XY (**a**); zirconia-sandblasted XY (**b**); corundum-sandblasted XY (**c**); as-built XZ (**d**); zirconia-sandblasted XZ (**e**); corundum-sandblasted XZ (**f**).

Figure 6 shows the evolution of the surfaces in the as-built condition and after both post-processing routes of the samples, fabricated along the XY and XZ orientations. The as-built surfaces exhibited the typical features of LPBFed surfaces, which can be summarized as follows: The XY surface was smoother and presented evident traces of the laser passes, while no partially adhered particles were present. Conversely, the XZ surface was rougher and had many partially adhered particles (Figure 6a–d). Both sandblasting techniques, which rely on mechanical energy, induced the compression of titanium alloy particles adhering to the surface and the creation of dimples in flatter areas of the surface, as evident in Figure 6b,c,e,f. In this respect, it is noted that partially adhering particles are among the main causes of dimensional deviation between CAD models and fabricated parts in lattice structures. 

The complex arrangement of the struts in the Voronoi lattice-based specimens dictates that the effect of sandblasting should be directly investigated. Firstly, a qualitative SEM evaluation of the surface appearance was carried out, and then a quantitative assessment of the dimensional deviation between the CAD models and the fabricated samples was performed to characterize these irregular lattice structures, which can be incorporated into orthopedic prostheses.

**Figure 7:**
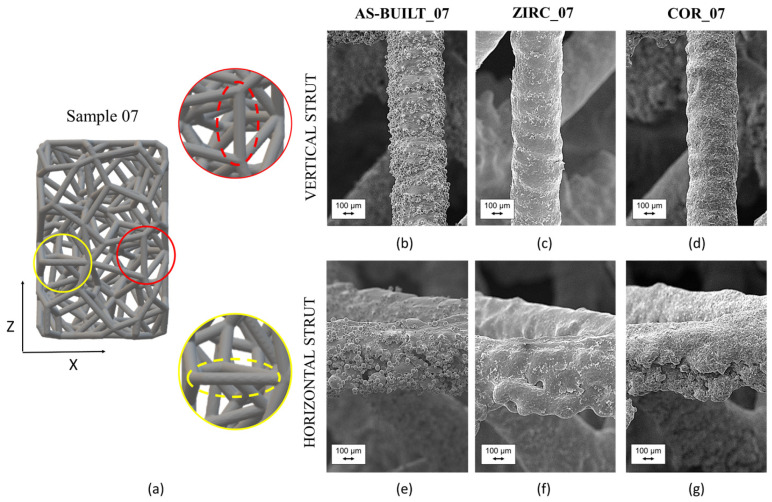
CAD model of sample 07 with close-up of vertical and horizontal struts (**a**); SEM of vertical strut in AS-BUILT_07 sample (**b**); SEM of vertical strut in ZIRC_07 sample (**c**); SEM of vertical strut in COR_07 sample (**d**); SEM of horizontal strut in AS-BUILT_07 sample (**e**); SEM of horizontal strut in COR_07 sample (**f**); SEM of horizontal strut in ZIRC_07 sample (**g**).

SEM observation of the struts in the Voronoi lattice-based samples subjected to different surface finishing techniques revealed that the lower skin and upper skin surfaces differed mainly in the horizontal struts, while the surface was understandably axially symmetric in the vertical ones (Figure 7). This is due to the nature of the LPBF process itself [38]. Tilted struts lay between these two extreme orientations (vertical and horizontal orientation). Particularly, the struts with axes tilted at less than 45° were more prone to warping than struts with more vertical axes (angle axis > 45°) [38]. Both sandblasting techniques modified the morphology of the horizontal and vertical struts (Figure 7c,d,f,g). For horizontal struts, it can be assumed that fatigue fractures may begin to occur near the evident discontinuities in the lower skin surface. 

Quantification of the process accuracy required a detailed evaluation of the dimensional deviation to be performed starting from the volumetric μ-CT reconstructions of the sandblasted samples. Thereafter, in order to isolate the effect of post-processing, the impact of different surface finishingsfinishing at an equal strut thickness (i.e., 0.7 mm) was addressed (AS-BUILT_07 vs. ZIRC_07 vs. COR_07).

Among the previously considered surface finishing techniques, zirconia sandblasting was the best choice for minimizing the dimensional deviation.

**Figure 8:**
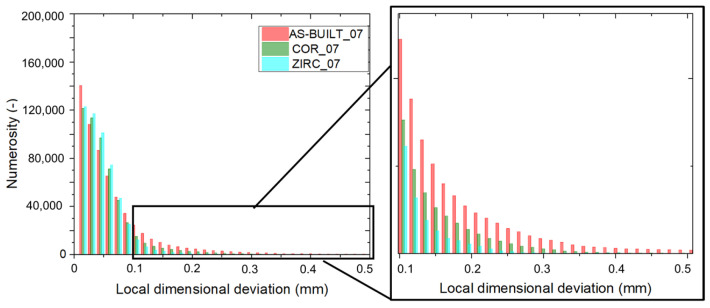
Histogram of dimensional deviations in the samples with a strut thickness of 0.7 mm in three difference surface conditions. Close-up of the region with local dimensional deviation > 0.1 mm.

**Figure 9:**
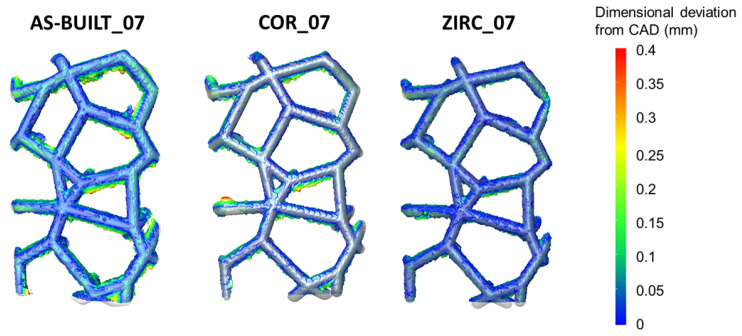
Comparison of the dimensional deviation values at a fixed thickness (0.7 mm) with varying surface conditions. Zirconia sandblasting provided a surface more similar to the CAD volume and helped reach the lowest dimensional deviation.

These results are quantitatively supported in Figure 8 and qualitatively evident in Figure 9. Qualitatively, the as-built surface in the ROI appeared very rough, and after corundum sandblasting, a fishbone pattern was noted, which was not evident in the zirconia-sandblasted samples (Figure 9). Due to the highly different morphologies observed in the lattice structures and their struts at different inclinations, at equal strut thickness, different surface finishing techniques led to different outcomes on the resulting surfaces. Zirconia sandblasting removed almost all the lower-skin warping, with a beneficial impact on the overall dimensional deviation (Figure 8). Indeed, the agglomerates evident in the as-built and corundum-sandblasted samples almost disappeared in the zirconia-blasted sample. This result was even more evident when the dimensional deviation was greater than 0.1 mm (zoom in Figure 8). 

Finally, regarding the roughness of the surface, zirconia sandblasting smoothed it out. 

It is possible to state that, regardless of the strut thickness, zirconia sandblasting resulting in the best surface finish to minimize dimensional deviation in the lattice structures, such as in the Voronoi lattice-based structures.

### 3.3. Two-Dimensional Global and Local Mechanical Behaviors

**Figure 10:**
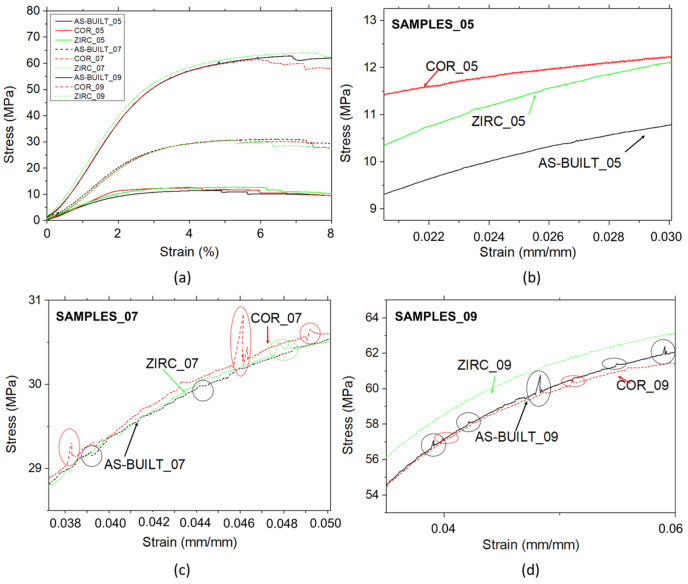
Stress–strain curves of all the samples (**a**); close-up of sample_05 (**b**), sample_07 (**c**), sample_09 (**d**). Circles highlight the up and down peaks in the stress–strain graphs.

Quasi-static compression tests were conducted to up to 8% of the global strain to prevent the failure of the struts. The global compression behavior of the lattice structures (Figure 10) showed that, as expected, the greater the thickness, the greater the maximum stress and the apparent Young’s modulus. The few drops in the stress–strain curves of sample_05 (both in the finished and as-built conditions) occurred at a greater strain as the thickness of the struts increased. The few drops were related to the initial failure of some thin struts, which were more stressed than others. This may in turn have been caused by the presence of defects, which had a stronger impact on the thinner struts.

As observed in the close-ups in Figure 10b–d, the zirconia-sandblasted samples exhibited smoother and more regular overall behavior than the as-built and corundum-sandblasted samples. These observations were more evident as the thickness of the struts increased. When the strut thickness was 0.5 mm, the samples presented similar behavior regardless of the surface finishing, and no evident peaks were detected (Figure 10b). On the contrary, many upward and downward peaks are evident in the graphs for sample_07 and sample_09 for the as-built and corundum-sandblasted samples (Figure 10c,d). These irregularities may be a consequence of the different surface morphologies, which led to a more irregular local mechanical behavior. 

Indeed, as observed in the µ-CT reconstructions (Figure 7), the zirconia-sandblasted surface was smoother than the others, and this resulted in a more regular trend in the stress–strain behavior (few peaks or no peaks). In contrast, the as-built samples and corundum-sandblasted samples, which presented rougher struts, showed more irregular behavior with up and down peaks in the stress–strain curves. At an equal thickness (e.g., 0.7 mm) and different surface finishes (as-built vs. corundum-sandblasted vs. zirconia-sandblasted), although the global behavior was similar among the different types of samples (i.e., similar maximum stress and similar young modulus), the trend of mechanical behavior differed. 

In conclusion, as confirmed by the μ-CT analyses, zirconia-sandblasting provided a smoother surface, which in turn produced more regular mechanical behavior.

The Young’s modulus and yield stress of the fully dense LPBFed Ti6Al4V ELI were 100 GPa and 850 MPa, respectively [6]. The designed lattice structures aimed to minimize the Young’s modulus with a beneficial impact on stress shielding during the interaction with bone tissue. 

**Figure 11:**
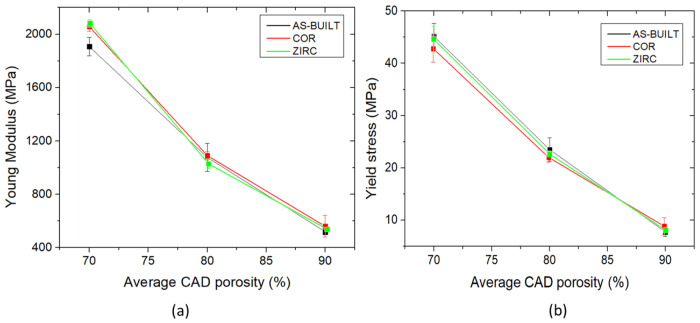
Correlations between the Young modulus and average CAD porosity (**a**), and the yield stress and average CAD porosity (**b**).

The dependance of the apparent Young’s modulus and the yield stress on the average CAD porosity in the Voronoi lattice structures is reported in Figure 11. As expected, a growing linear trend was observed in both graphs. The lower the average porosity, the stiffer and more resistant the entire lattice structure. As depicted in Figure 11a, sandblasted samples were stiffer than the as-built samples, regardless of the strut thickness: nevertheless, no statistical differences among the samples with similar strut thicknesses and different surface finishings were observed. The apparent Young’s modulus values were compatible with the bone modulus (0.5–2 GPa), confirming that these structures, regardless of the surface finish, could be integrated into bulk prostheses, minimizing the possible stress-shielding effect [2,39]. 

From these preliminary results, it is possible to state that, as shown in Figure 11a,b, the global mechanical response of the Voronoi lattice-based samples was not strongly affected by the different surface finishings. However, it was evident that zirconia sandblasting decreased the overall standard deviation in the experimental tests and led to more repeatable and reliable results for the mechanical behavior of such complex structures.

Since no evident global differences were observed, and considering that surface finishing is a technique that leads to local changes, further analysis of the local mechanical behavior as a function of strut thickness and surface condition were carried out. 

**Figure 12:**
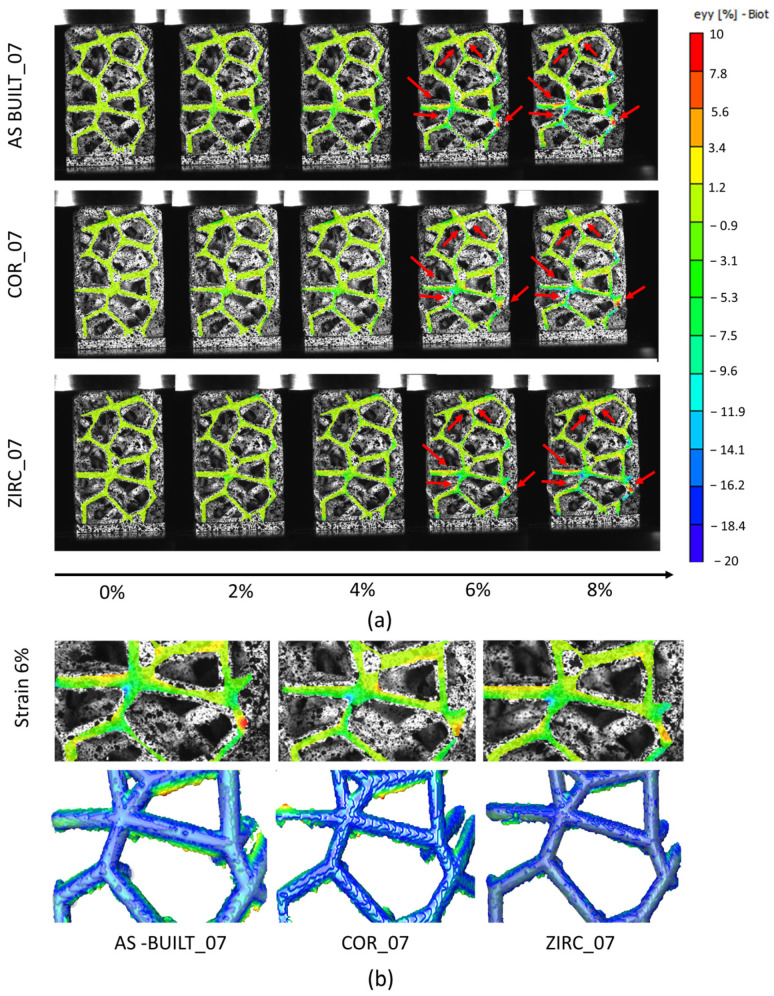
DIC results for sample_07 at different strain values. Red arrows indicate the points with higher local strain values (**a**); comparison of DIC results at 6% of strain and dimensional deviation from μ-CT (**b**).

The DIC analysis provided useful quantitative indications on the local strain distributions in the ROI highlighted in Figure 3. Figure 12 shows the results for sample_07 under the three surface conditions (as-built vs. corundum-sandblasted vs. zirconia-sandblasted). Similar results characterized sample_05 and sample_09, regardless of the surface finishing. While the local strain maps showed the same Ɛyy values regardless of the surface conditions up to 4% of the global strain, at 6% and 8% of the global deformation, the as-built sample revealed areas of local strain intensification, which are pointed out in Figure 12a by red arrows. The main, small differences were evident at the nodes, where possibly powder particles accumulated and were then removed during the surface finishing. To verify whether the small differences in the local strain maps could be related to the previously assessed dimensional deviations, a comparison between the DIC results and the μ-CT models was performed (Figure 12b). Despite the evident dimensional deviation, the differences in the local strain maps were more likely related to the different ways of producing the samples rather than to the surface finishing and the consequent dimensional deviation. Indeed, the main differences in local strain maps did not occur where the dimensional deviation was present. It is likely that greater differences could be observed during fatigue tests, on which, as is well established, the surface finishing process has a significant impact.

## 4. Conclusions

The current research aimed to assess the impact of surface finishing on dimensional deviation in Voronoi samples with different strut thicknesses and to evaluate and identify differences in local strain maps due to the applied surface finishes. 

Realizing and post-processing complex structures fabricated by AM, while accurately reproducing the CAD geometry, is crucial in order to obtain a reliable outcome. Moreover, it is well established that the more complex the structure, the more difficult its fabrication and post-processing (e.g., the surface finishing of lattice structures). 

Moreover, the dimensional deviation of a part fabricated in AM should be accurately assessed, as possible discrepancies may induce deviations between computational results and experimental tests. Instead of using the μ-CT .stl file of the part fabricated in AM, which is often difficult to manage due to its dimensions, the knowledge of the dimensional deviation could allow for the preparation of a proper CAD model, which can be used for the FEA and providing a reliable comparison with experimental tests.

Finally, the huge interest in AM processes in the biomedical field and the evident interest in the design and fabrication of lightweight lattice structures makes the current research helpful for the improvement and refinement of 3D components.

The current research demonstrated that the surface finishing strongly affects both simple discs’ morphologies (i.e., 2D geometry) and Voronoi lattice-based surfaces (i.e., 3D geometry). Focusing on the Voronoi lattice-based structures, under equal surface conditions, the greater the strut thickness, the greater the number of points among which the dimensional deviation is small. The smaller the struts thickness, the greater the number of points among which the dimensional deviation is large. Furthermore, the processability and quality of samples could be strongly affected by the inclinations of the struts. At an equal strut thickness (e.g., 0.7 mm), zirconia sandblasting led to the lowest dimensional deviation, making it the most interesting surface finishing in view of minimizing the dimensional deviation in complex structures.

Concerning the global mechanical behavior, it was observed that zirconia sandblasting resulted in smoother behavior.

The DIC results show that, at an equal strut thickness, different surface finishings did not induce evident differences in the local strain maps. The few highlighted differences were ascribed to the different surface morphologies; however, no general or statistically significant conclusion can be drawn due to the low number of observed differences.

Further studies are ongoing for the assessment of the impact of strut thickness and surface finishing on fatigue resistance.

In conclusion, the current research provides useful indications on the surface finishing to adopt according to the intended application of the addressed structures in the biomedical field.

## Figures and Tables

**Table 1:** Sample codes definitions.

Sample	Features	Surface Condition	Code	Numerosity of Replicas
Sample_05	Strut thickness of 0.5 mm	As-built	AS-BUILT_05	3
		Corundum-sandblasted	COR_05	3
		Zirconia-sandblasted	ZIRC_05	3
Sample_07	Strut thickness of 0.7 mm	As-built	AS-BUILT_07	3
		Corundum-sandblasted	COR_07	3
		Zirconia-sandblasted	ZIRC_07	3
Sample_09	Strut thickness of 0.9 mm	As-built	AS-BUILT_09	3
		Corundum-sandblasted	COR_09	3
		Zirconia-sandblasted	ZIRC_09	3

## Data Availability

The data presented in this study are available on request from the corresponding author. The data are not publicly available due to privacy restrictions.

## Abbreviations

LPBF, laser powder bed fusion; DIC, digital image correlation; AM, additive manufacturing; CAD, computer-aided design; μ-CT, micro-computed tomography; XY, parallel samples to the building direction; XZ, perpendicular samples to the building direction; Ti6Al4V ELI, extra-low interstitial; SEM, scanning electron microscopy; AS-BUILT, sample with surface in as-built condition; COR, sample with corundum-sandblasted surface; ZIRC, sample with zirconia-sandblasted surface; (…)_05, sample with a strut thickness of 0.5 mm; (…)-07, sample with a strut thickness of 0.7 mm; (…)_09, sample with a strut thickness of 0.9 mm; ASTM, American Society for Testing and Materials.
